# Mirizzi Syndrome Type I: A Case Presentation

**DOI:** 10.7759/cureus.37029

**Published:** 2023-04-02

**Authors:** Michelle N Won, Dylon P Collins, Stephanie Bouchard, Christopher Finley

**Affiliations:** 1 Dr. Kiran C. Patel College of Osteopathic Medicine, Nova Southeastern University, Fort Lauderdale, USA; 2 Bariatric and Minimally Invasive Surgery, Fawcett Florida Memorial Hospital, Port Charlotte, USA

**Keywords:** obstructive jaundice, cholecystectomy, gallbladder, cholelithiasis, mirizzi syndrome

## Abstract

Mirizzi syndrome (MS) is a rare complication of chronic cholelithiasis. The syndrome describes gallstone obstruction of Hartmann’s pouch or the cystic duct that extrinsically compresses the common hepatic duct, causing obstructive jaundice. In advanced cases, the gallstones may erode into the biliary tree creating a fistula, requiring prompt diagnosis and careful surgical management. We present a case of an 82-year-old female who presented with upper abdominal pain and jaundice, later diagnosed with suspected MS type I, and managed surgically. We aim to highlight MS type I because of the potential progression and damage to the bile duct, creating complications that may affect overall patient outcome.

## Introduction

The gallbladder is a pear-shaped organ attached to and located on the inferior right lobe of the liver and, when fully distended, measures roughly 7 x 4 cm. Bile is formed in the liver and stored in the gallbladder. Hardening bile in the gallbladder leads to the formation of gallstones, also known as cholelithiasis. There are three methods by which gallstones form: cholesterol supersaturation, excess bilirubin, and gallbladder hypomotility or impaired contractility. Cholelithiasis is relatively common, found in approximately 6% of men and 9% of women in the United States [1]. In some instances, gallstones can grow so large that they become impacted in the gallbladder’s Hartmann’s pouch or cystic duct, causing external compression on the common hepatic duct (CHD). This condition is known as Mirizzi syndrome (MS), a rare complication of cholelithiasis.

MS was first described in the early 1900s and later named in 1948 by physician Pablo Luis Mirizzi [2]. MS describes the impaction of gallstones in the neck of the gallbladder that erodes into the bile duct causing obstructive symptoms. The incidence has been estimated to fall between 0.7% and 2.9% of all cholecystectomies [2]. In chronic cholelithiasis, the stones may ulcerate and create a cholecystocholedochal or cholecystohepatic fistula. Therefore, it is essential to be aware of MS because of the complications that can arise with bile duct injury. Here, we present a case of an 82-year-old female suffering from unrelievable epigastric pain and obstructive biliary symptoms who underwent definitive treatment with a cholecystectomy due to suspected MS type 1.

## Case presentation

An 82-year-old caucasian female presented to the emergency department at a local hospital with upper back and upper abdominal pain. Her past medical history consisted of diabetes and obesity, both resolved by gastric bypass surgery eight years prior to her presentation. She had previous epigastric pain that was relieved by antacids. However, this pain was constant and unremitting. She denied vomiting, fever, and lower abdominal pain. On examination, she was found to have jaundice with localized right upper quadrant tenderness and a positive Murphy's sign.

Pertinent labs on admission were increased total bilirubin, direct bilirubin, aspartate aminotransferase (AST), alanine aminotransferase (ALT), alkaline phosphatase and lipase, and a normal level of WBCs (Table 1).

**Table 1:** Patient lab values AST: aspartate aminotransferase, ALT: alanine aminotransferase

	Value	Reference range
Total bilirubin (pre-surgery)	5.38 mg/dL	0.2-1.0 mg/dL
Total bilirubin (post-surgery)	1.76 mg/dL	0.2-1.0 mg/dL
Direct bilirubin	4.64 mg/dL	0.0-0.20 mg/dL
AST	59 U/L	15-37 U/L
ALT	87 U/L	12-78 U/L
Alkaline phosphatase	319 U/L	45-117 U/L
Lipase	462 U/L	73-393 U/L
WBCs	6.5 U/L	4-10.5 U/L

**Figure 1:**
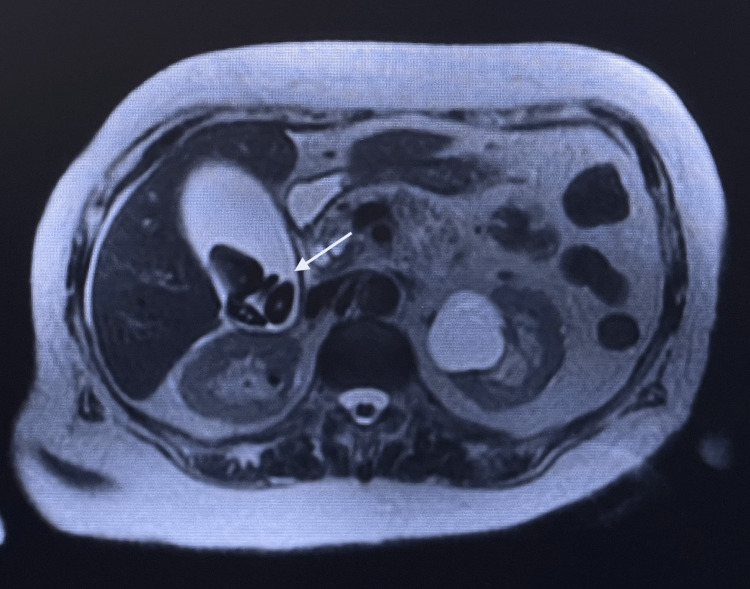
MRCP showing large triangular gallstones contained within the gallbladder

A CT scan of the abdomen and pelvis with contrast showed a distended gallbladder with multiple noncalcified gallstones. There was no wall edema or pericholecystic fluid and no intrahepatic or extrahepatic biliary ductal dilation. A magnetic resonance cholangiopancreatography (MRCP) was utilized and confirmed a distended gallbladder with cholelithiasis and demonstrated no biliary filling defect, dilation, or strictures (Figure 1). The patient was referred to general surgery for laparoscopic cholecystectomy.

**Figure 2:**
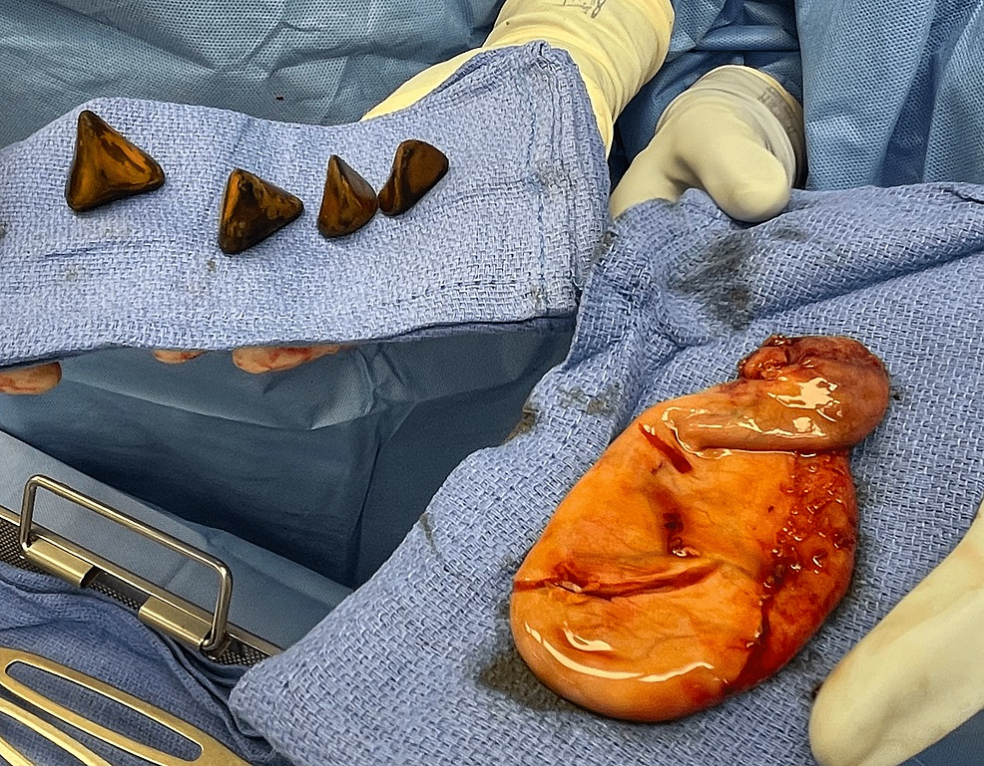
Postoperative gallbladder and gallstones

**Figure 3:**
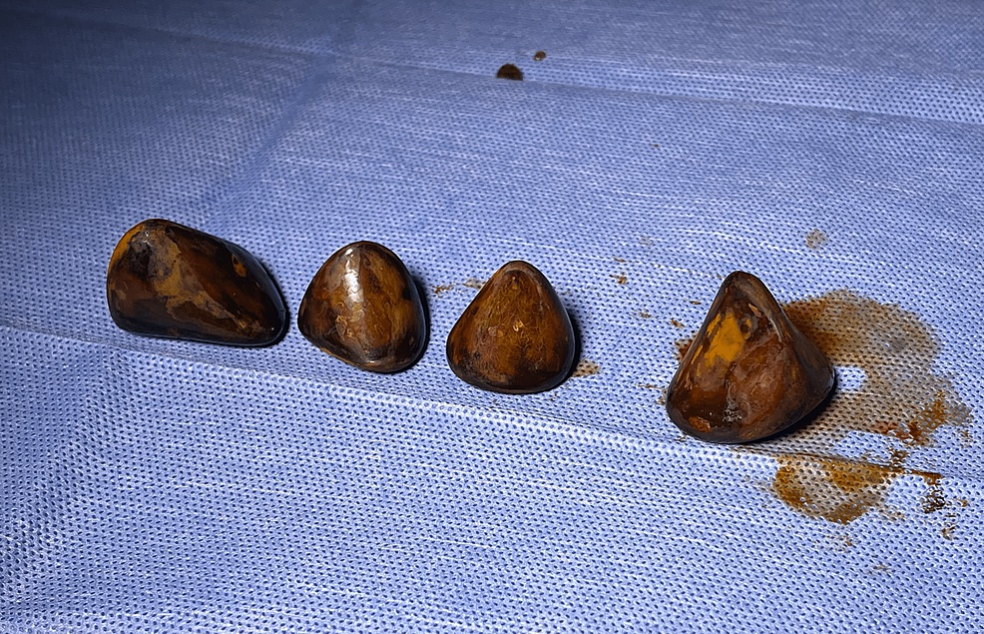
Four large gallstones removed from the gallbladder post-operatively

A markedly enlarged gallbladder was visualized during the surgery, 100 ccs of bilious fluid were drained from the gallbladder, and the head of the gallbladder was floppy and found to be impacted with large stones. A critical view of safety was obtained. The cystic duct and artery were clipped and transected, and the gallbladder was removed using an Endo Catch bag through the 12 mm umbilical port with great difficulty due to the size of multiple stones. Postoperatively, the gallbladder, measuring 10.2 x 5 x 3.4 cm, was opened and four large, brown, triangular calculi were present, with the largest measuring 3.4 cm in greatest dimension (Figures 2, 3).

No polyps or masses were observed. Labs were drawn the following day, and the patient’s total bilirubin had significantly decreased (Table 1). The patient was discharged after her post-acute care recovery. At her one-week follow-up, she had no complaints with complete resolution of symptoms, and the histology reports showed no signs of malignancy.

## Discussion

Initially, McSherry et al. broadly classified MS into type I and type II [3]. Type I, also considered classic MS, demonstrates external compression of the common bile duct or CHD, and Type II describes the formation of a cholecystobiliary fistula. Csendes proposed the currently accepted classification system of MS in 1989, which further divided type II by the extent of damage and erosion into the common bile duct [4]. Type II lesions form a cholecystobiliary fistula with erosion into the bile duct less than one-third of its circumference. In type III lesions, the fistula involves up to two-thirds of the duct circumference, and type IV lesions show complete destruction of the bile duct.

Common symptoms of biliary obstruction are jaundice, fever, and right upper quadrant pain, but most patients do not present with all three. MS is often diagnosed intraoperatively and postoperatively due to similar imaging findings and lab results as other causes of obstructive jaundice, such as cholelithiasis, choledocholithiasis, or biliary strictures. Additionally, an extrahepatic tumor, such as a Klatskin tumor, may show similar clinical signs and stricture findings as MS. The surgical management of MS requires an experienced hepatobiliary surgeon, as the bile ducts may be injured if a cholecystobiliary fistula is present. Preoperative diagnosis has decreased the risks of open conversion, procedure-related complications, and reoperation compared to a low preoperative diagnosis [5]. Understanding the clinical signs, symptoms, and operative findings of MS is crucial to avoiding a diagnostic error.

Preoperative imaging, such as ultrasound, CT, endoscopic retrograde cholangiopancreatography (ERCP), and MRCP, are often essential to differentiate the cause of the obstructive process [6]. MRCP was utilized in this patient. ERCP is considered the gold standard for diagnosing MS due to its high sensitivity rate of 76.2% [7]. Furthermore, ERCPs are not only diagnostic but also considered therapeutic because of the ability to visualize fistula formation and place biliary stents to alleviate obstructive symptoms [7]. An ERCP was not feasible as the patient had altered anatomy due to a previous gastric bypass. A cholangiogram was unavailable due to the constraints of a small community hospital setting. However, prior to surgery, biliary obstruction was ruled out on CT and MRCP.

The case presented is highly suspicious of MS type I due to the large size of multiple gallstones, obstructive signs and symptoms-elevated direct bilirubin and jaundice, and resolution postoperatively. Additionally, CT and MRCP revealed patent common hepatic and common bile ducts, ruling out choledocholelithiasis. Although the patient did not present with an expected dilated CHD, this case demonstrates suspected MS before it reaches chronic inflammatory compression leading to erosion and fistula formation into the bile duct.

The mainstay of definitive treatment for MS is surgery. Laparoscopic cholecystectomies are commonly performed on patients with MS type I, which resolves the biliary duct compression and inflammation. However, the risks for open conversion and surgical complications are similar for type I and type II [5]. If MS is discovered intraoperatively, a cholangiogram is needed during surgery to assess the extent of biliary damage. Commonly, surgeons will find anatomical difficulties during the dissection of Calot's triangle due to extensive inflammation and large stone compression. In MS types II-IV, surgical procedures, such as choledochoplasty with gallbladder flap and T-tube placement or a preferred bilioenteric anastomosis for complex bile duct erosion, are often required [8]. Overall, this further illustrates why preoperative suspicion and diagnosis are crucial for optimal surgical management and outcome.

## Conclusions

MS type I is typically benign due to transient compression of the bile duct, but chronic compression can lead to erosion and fistula formation. It is imperative to have raised suspicion in patients presenting with obstructive jaundice and to investigate its causes adequately. Early diagnosis of MS will lead to the best surgical and post-surgical outcomes.
